# Impact of COVID-19 pneumonia on pulmonary vascular volume

**DOI:** 10.3389/fmed.2023.1117151

**Published:** 2023-03-22

**Authors:** Guillaume Fahrni, Ana-Carolina Rocha, Louis Gudmundsson, Chiara Pozzessere, Salah D. Qanadli, David C. Rotzinger

**Affiliations:** ^1^Cardiothoracic and Vascular Division, Department of Diagnostic and Interventional Radiology, Lausanne University Hospital and University of Lausanne, Lausanne, Switzerland; ^2^Riviera Chablais Hospital and University of Lausanne, Lausanne, Switzerland

**Keywords:** COVID-19, vascular volume, anastomoses, venous congestion, arteriovenous shunt, CT, imaging

## Abstract

**Background:**

Pulmonary manifestations of COVID-19 pneumonia are well known. However, COVID-19 is also associated with a range of vascular manifestations such as embolism, congestion, and perfusion changes. Regarding congestion, research from different groups has suggested arteriovenous anastomosis dysregulation as a contributing factor. In this study, we aim to better describe the changes in vascular volume in affected lung zones and to relate them to pathophysiological hypotheses.

**Methods:**

We performed automatic vascular volume extraction in 10 chest CTs of patients, including 2 female and 8 male with a mean age of 63.5 ± 9.3 years, diagnosed with COVID-19 pneumonia. We compared the proportion of vascular volumes between manually segmented regions of lung parenchyma with and without signs of pneumonia.

**Results:**

The proportion of vascular volume was significantly higher in COVID (CVasc) compared to non-COVID (NCVasc) areas. We found a mean difference (DVasc) of 5% and a mean ratio (RVasc) of 3.7 between the two compartments (*p* < 0.01).

**Conclusion:**

Vascular volume in COVID-19 affected lung parenchyma is augmented relative to normal lung parenchyma, indicating venous congestion and supporting the hypothesis of pre-existing intra-pulmonary arteriovenous shunts.

## 1. Introduction

While the computed tomography (CT) pulmonary manifestations of the Coronavirus disease 2019 (COVID-19) have been widely described (1–6), vascular manifestations have been less frequently reported and remain poorly understood. Multiple vascular changes are described in the literature, including mainly pulmonary embolism (7–10), vascular congestion or enlargement (11–17), and perfusion changes (18–21). Until now, the link between morphological changes and the correlation between biological changes and virus-induced inflammatory disorders remains an area of research. Furthermore, the COVID-19 acute respiratory distress syndrome (ARDS) is different from a typical pre-COVID ARDS, with relatively preserved lung volumes compared to the state of respiratory failure (22–24). Those findings suggest that a ventilation-perfusion mismatch phenomenon is not entirely responsible for the pathophysiology, but other vascular changes are implied in the process.

In this work, we investigate the blood volumes between healthy (COVID-19-free parenchyma) and diseased (COVID-19 alveolar opacity) lung regions in patients with COVID-19 pneumonia subjected to a computed tomography angiography (CTA). Our hypothesis is that the activation of arteriovenous anastomoses in affected lung regions under hypoxic conditions may translate into an augmented regional vascular volume on imaging.

## 2. Materials and methods

### 2.1. Patients

Patients were recruited from the Swiss national registry coronavirus-associated vascular abnormalities (COVID-CAVA; clinicaltrials.gov identifier NCT04824313), a multicentric cohort of patients who underwent chest CT and had microbiologically-proven COVID-19 infection, with the aim to assess non-vascular and vascular findings (25). In this cohort, we already demonstrated that vascular congestion observed in COVID-19 primarily affects veins, but without quantitatively comparing the fraction of vascular components in affected vs. non-affected lung segments (17). We consecutively included 24 patients from the 12th of March to the 28th of April 2020 from the Lausanne University Hospital Center (CHUV), Lausanne, Switzerland. The exclusion criteria were: absence of ground glass opacities (GGO) changes related to COVID-19 (*n* = 0), presence of excessive parenchymal consolidation (*n* = 14), and absence of other pulmonary parenchymal or vascular disease (*n* = 0). Excessive consolidation was defined as more than 20% of COVID-19 parenchymal zones affected with consolidations instead of GGO. Patients with excessive parenchymal consolidation were excluded because threshold-based vascular compartment segmentation was not achievable as it resulted in inaccuracies because a substantial part of parenchymal consolidation would be segmented as vascular volume, even with thresholds adjusted towards higher CT numbers. Patients with other pulmonary vascular or parenchymal disease were excluded so that only pure COVID-related vascular volume changes could be analyzed. We collected patient’s age, sex, symptoms, type of care (ambulatory, medical ward [MW], intensive care unit [ICU]), oxygenation and laboratory findings (D-Dimers, pO2, and CRP) in the electronic medical record system. The local Ethics Committee approved the protocol, and patient consent was waived.

### 2.2. CT protocol

CT examinations were performed using a multidetector fast kV-switching dual-energy CT scanner (Revolution CT, GE Healthcare, Milwaukee, WI, United States), with a 0.23 mm spatial resolution. Images were acquired with the following parameters: rotation speed, 0.5 s; voltage, 80/140 kVp; tube load, 249 to 485 mAs; reconstructed slice thickness, 1.25 mm; and section interval, 1 mm; 80 keV. 60 to 100 mL (depending on patient’s size and weight at the discretion of the radiology technician in charge) of iodinated contrast material (Accupaque 300^®^, GE Healthcare, Oslo, Norway) were injected intravenously followed by a saline chaser (17, 25). All exams were acquired to enhance both pulmonary and systemic vessels, using the bolus tracking technique to trigger the acquisition as per routine protocols applied in the department.

### 2.3. Semi-automatic lung volumes segmentation

All segmentations were performed using AW-server software (version 3.2, GE Healthcare, Buc, France). First, total lung volumes were extracted using automatic segmentation. This process isolated the voxels related to lung parenchyma and vessels from the rest of the chest. It included pulmonary arteries and vein branches but not the proximal vessels. Lungs were separated into left and right lungs, each with a corresponding extracted lung volume.

Each lung was then separated into healthy and COVID pneumonia volumes. COVID-19-related alveolar opacification volumes were quantified using computer-aided manual extraction, with manual contour segmentation on approximately every 10 slices and automatic interpolation between slices. Healthy volumes were then calculated by subtracting the COVID lung volume from the total lung volume.

### 2.4. Vascular volume extraction

**Figure 1:**
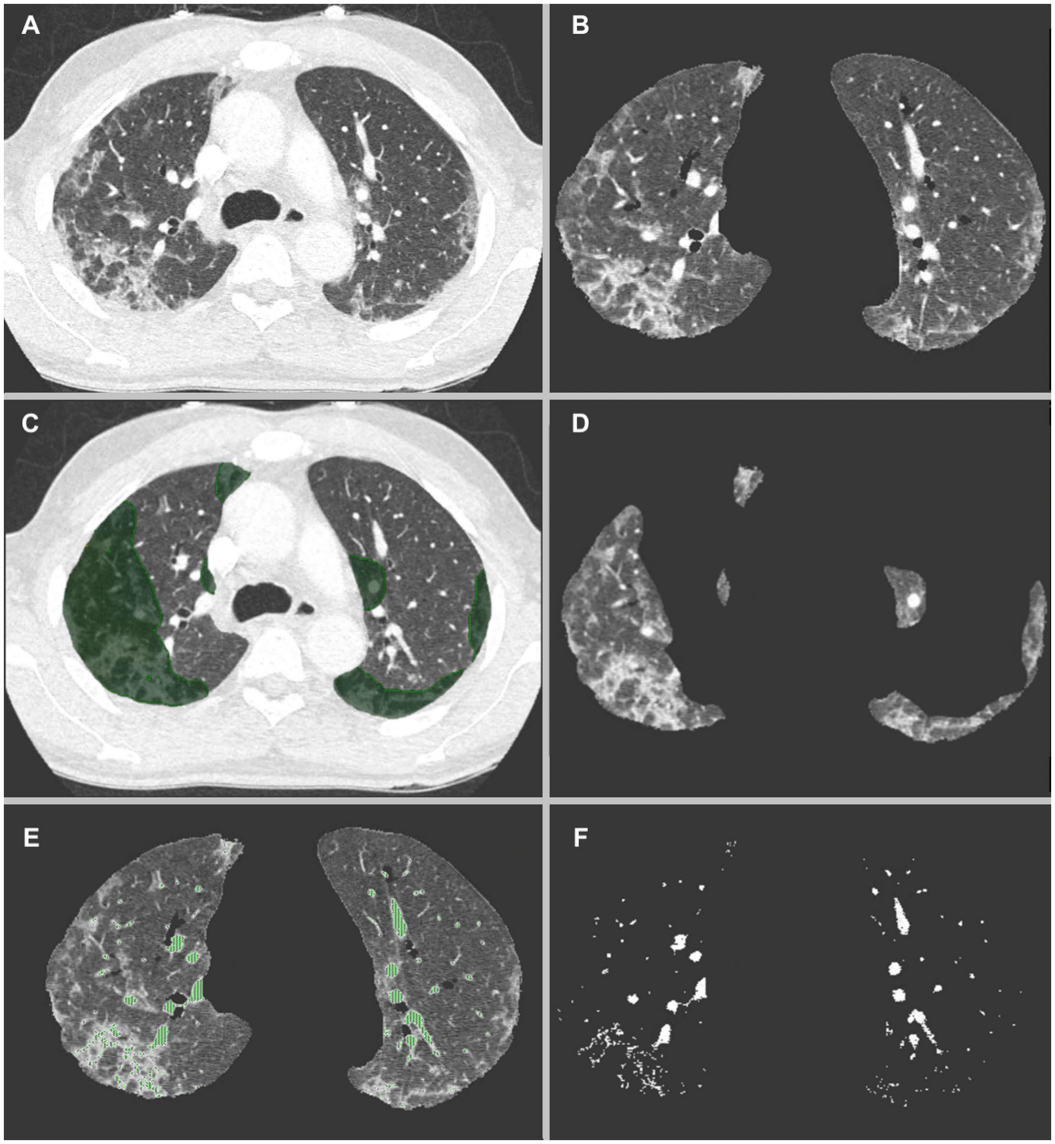
**(A)** CT-scan of a COVID-19 patient showing multifocal zones of group glass opacities. **(B)** Automatic lung volumes extraction. **(C)** Manual segmentation of COVID-19 volumes, keeping only the parenchymal part of the segmentations. **(D)** Resulting segmented COVID-19 volumes, and **(E)** Automatic HU threshold based vascular volumes extraction. In this patient a threshold of −50 HU was used. Venous congestion as well as increased distal vascular volume is seen in the regions affected by COVID-19 pneumonia **(F)**.

Vascular volumes were extracted using CT number thresholds to isolate voxels containing vascular elements. Because of interpatient variability, the CT number threshold (in Hounsfield Units [HU]) was contrast enhancement-dependent and manually defined, in order to separate as much as possible densities corresponding to vascular structures, i.e., pulmonary arteries, pulmonary veins and to a lesser extent systemic arteries which account for a lower portion of the vessels as previously demonstrated (17), from the densities corresponding to parenchyma. Figure 1 summarizes the lung volume and vascular segmentation processes.

The same threshold was used in each patient to extract vascular volume in the total lung volumes, the COVID lung volumes, and the right and left lobes. Vascular volumes in non-affected parenchyma were calculated by subtracting COVID vascular volume from the total vascular volume.

### 2.5. Volumes analysis

For each patient, we defined the following volumes: total lung volume (TLV), total COVID lung volume (TCV) representing alveolar opacification including both consolidation and ground glass opacity, total non-COVID lung volume (TNCV), total vascular volume (TVV), COVID vascular volume (CVV) representing vascular components in lung tissue affected by alveolar opacification and non-COVID vascular volume (NCVV). We then calculated and compared the proportion of vascular volumes within the COVID (C_VASC_ = CVV/TCV) and non-COVID (NC_VASC_ = NCVV/TNCV) lung parenchyma, which ultimately reflects the difference in vascular volume between healthy and diseased zones in the lung. Comparisons were performed in terms of difference (D_vasc_ = C_VASC_ – NC_VASC_) and ratio (R_Vasc_ = C_VASC_ / NC_VASC_).

Statistical analysis was conducted with Rstudio 4.1. Results were reported as the number of subjects and percentages. Continuous variables were tested for normality with the Shapiro–Wilk test and compared with a Wilcoxon rank-sum test, with a significance threshold of 0.05.

## 3. Results

Out of the 27 patients screened for inclusion, we excluded 17 due to excessive lung consolidation. We thus included 10 patients with images of predominant ground glass opacities. Patient’s characteristics are detailed in Table 1.

**Table 1:** Patient characteristics.

Characteristics	Patients (*n* = 10)
*Mean age ± SD (y)*	63.5 ± 9.3
*Sex, n (%)*	
Male	8 (80)
Female	2 (20)
*Type of care, n (%)*	
Ambulatory	1 (10)
Medical ward (MW)	4 (40)
Intensive care unit (ICU)	5 (50)
*Symptoms, n (%)*	
Fever	7 (70)
Dyspnea	10 (100)
Weakness	7 (70)
Myalgia	3 (30)
Cough	8 (80)
*Mean O2 therapy ± SD (L/min)*	3.7 ± 3
*Laboratory Findings ± SD*	
D-Dimers (ng/mL)^1^ [normal <500 ng/mL]	1,317 ± 791.4
pO2 (mmHg)	76.5 ± 25.4
CRP (mg/L) [normal <10 mg/L]	103.8 ± 103.2

1 Data available for 9 patients.

Computer-aided lung volume segmentation provided satisfactory results, with adequate delimitation between the lung and the non-lung structures. Total lung volumes (TLV) ranged from 1723 to 3,504 mL (mean: 3558 mL, ± 973 mL). Manually segmented total COVID volumes (TCV) ranged from 571 to 2,704 mL (mean: 1730 mL, ±748 mL). Subtracted total non-COVID volumes (TNCV) ranged from 451 to 2,849 mL (mean: 1829 mL, ±986 mL).

Vascular volume segmentation also provided adequate results with minimal overlap between lung parenchyma and vessels. Segmentation thresholds ranged from −10 to –150HU (mean: –65HU, ± 54HU). Extracted total lung vascular volumes (TVV) ranged from 71 to 388 mL (mean: 185 mL, ±93 mL). Extracted COVID vascular volumes (CVV) ranged from 49 to 314 mL (mean: 139 mL, ±82 mL). Subtracted healthy vascular volumes (NCVV) ranged from 3 to 79 mL (mean: 46 mL, ±27 mL).

**Figure 2:**
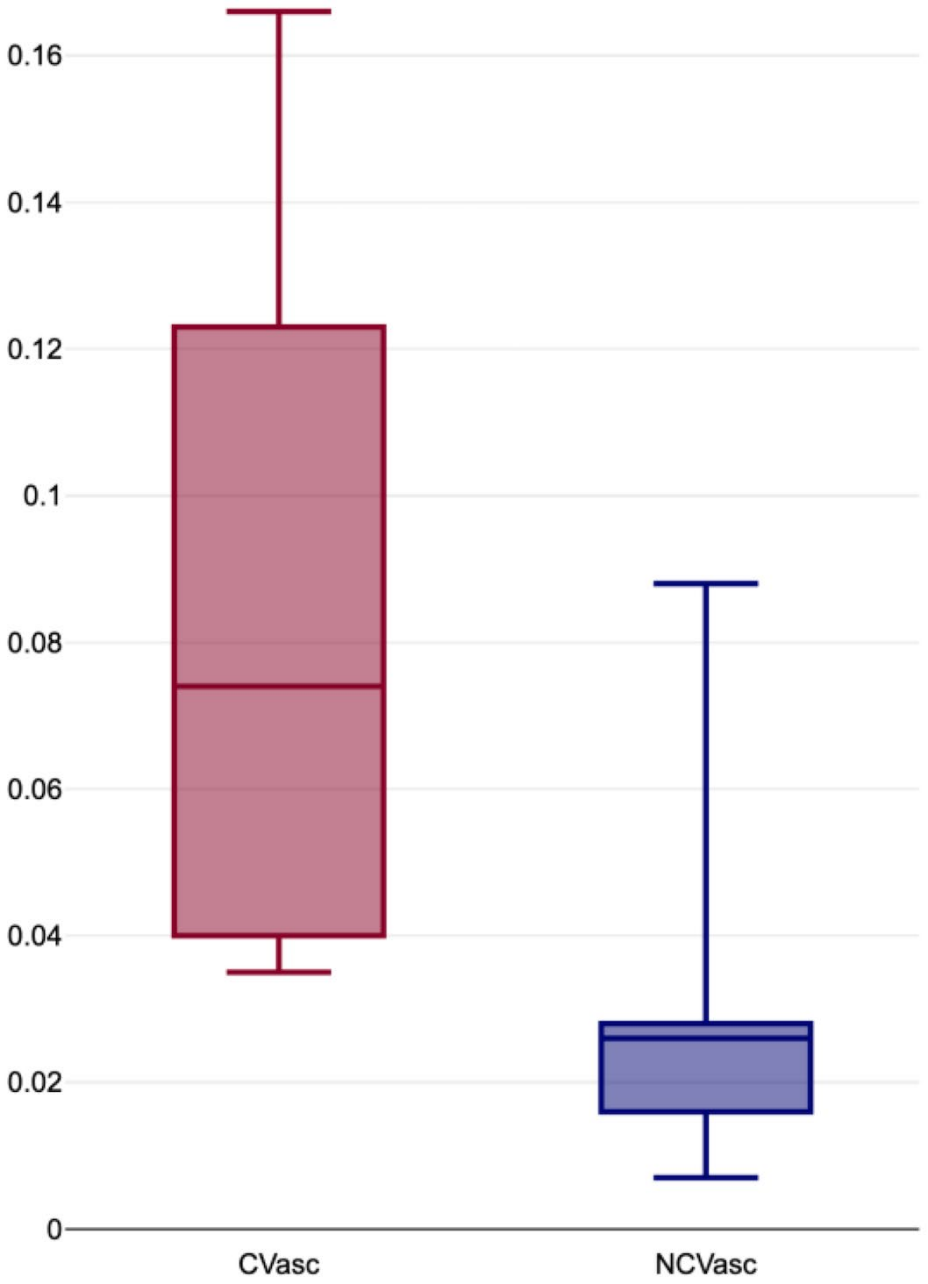
Boxplot distribution of proportion of vascular volumes within COVID-19 affected lung volumes (C_Vasc_) and normal lung volumes (NC_Vasc_).

The proportion of vascular volumes within diseased lung volumes (C_Vasc_) ranged from 4 to 17% (mean: 8, ±5%), while the proportion of vascular volume within non-COVID lung volumes (NC_Vasc_) ranged from 1 to 9% (mean: 3, ±2%) (Figure 2). C_Vasc_ was systematically higher than NC_Vasc_, with differences (D_Vasc_) ranging from 1 to 14% (mean: 5, ±4%) and ratios (R_Vasc_) ranging from 1.3 to 5.95 (mean: 3.7, ± 2.08). All results are displayed in Table 2.

**Table 2:** Total lung volume (TLV), total COVID volume (TCV), total non-COVID volume (NCV), COVID vascular volume (CVV), non-COVID vascular volume (NCVV), proportion of vascular volume in COVID areas (C_vasc_), proportion of vascular volume in non-COVID areas (NC_Vasc_), difference of vascular volume proportions between COVID and non-COVID areas (D_Vasc_), ratio of vascular volume proportions between COVID and health areas (R_Vasc_), in 10 COVID-19 patients.

Patient	TLV	TCV	TNCV	TVV	CVV	NCVV	C_Vasc_	NC_Vasc_	D_Vasc_	R_Vasc_
1	3,504	2,453	1,051	114	86	28	0.035	0.027	0.008	1.32
2	3,155	2,704	451	110	107	3	0.040	0.007	0.033	5.95
3	2,855	912	1943	71	34	37	0.037	0.019	0.018	1.96
4	2,850	1965	885	150	139	11	0.071	0.012	0.058	5.69
5	3,663	571	3,092	128	49	79	0.040	0.028	0.013	1.46
6	4,606	1957	2,649	388	314	74	0.166	0.027	0.139	6.10
7	4,062	1,213	2,849	220	149	71	0.123	0.025	0.098	4.93
8	4,072	2,216	1856	264	212	52	0.096	0.028	0.068	3.41
9	5,095	2,332	2,763	223	179	44	0.077	0.016	0.061	4.82
10	1723	972	751	188	122	66	0.126	0.088	0.038	1.43
mean	3,559	1730	1829	186	139	47	0.081	0.028	0.053	3.71
SD	973	748	986	93	82	27	0.046	0.022	0.041	2.08

Statistical analysis using a Shapiro–Wilk test showed a normal data distribution, however due to small sample size, a non-parametric test was used. Wilcoxon rank-sum test showed significant differences between C_Vasc_ and NC_Vasc_ (*p* = 0.026).

## 4. Discussion

In this study, we assessed the difference of vascular volumes between normal and affected lung parenchyma in COVID-19 patients. We found that affected zones showed significantly increased vascular volumes compared to normal zones. Vascular disorders in COVID-19 are still being investigated, and their distribution and prevalence are debated. Pulmonary embolism is one of the most researched topics related to vascular changes in COVID-19. In a multicenter study including 413 patients, Sadjad and al. found a pulmonary embolism incidence of 25% in hospitalized patients (7), while a systematic literature search identified in 27 studies with 3,342 patients a prevalence of 16.5% in all patients scanned for COVID-19 (9). Recently, Nevesny et al. (17) highlighted a potential connection between pulmonary embolism and venous congestion in the affected territories without arterial dilatation (17). More importantly, Nevesny et al. demonstrated that vascular congestion primarily happens in the venous compartment in COVID-19 pneumonia, with normal caliber veins in the non-affected zones, directing the discussion of pathophysiology towards specific mechanisms. The venous congestion they measured was at the segmental and subsegmental level, due to the difficulty to measure smaller vessels, which is something we also visually observed, but it could affect the whole venous tree and even the capillaries. Such venous congestion associated with paradoxical regional oligemia have raised the question of vascular shunting. Pre-existing, dysregulated arteriovenous anastomoses have been suggested as another phenomenon contributing to the vascular changes in COVID-19 (26–32). One theory is based on COVID-19 induced inflammation at the level of the capillaries, possibly inducing nitric oxide synthase (NOS) and nitric oxide (NO) production (33). This hypothesis describes increased NO activates precapillary arteriovenous anastomoses, usually present during fetal life and partially regressing after birth, leading to a right to left shunt and thus causing dead spaces and worsening hypoxemia (26, 30–32). This hypoxemic cascade explains the discrepancies between severe respiratory failures despite relatively moderate ventilation loss (22). More recently, Ackermann et al. demonstrated increased bronchopulmonary shunting, in other words arteriovenous anastomoses between bronchial arteries and pulmonary veins in hypoxic COVID-19 lung area, based on dual-energy CT, phase-contrast tomography, histology, and scanning electron micrography (34). Another hypothesis would be that venous obstruction or constriction would induce upstream microvascular congestion and elevated vascular pressure, leading to A-V shunts recruitment. While we did not observe this at the venous level, it could affect small veins, venules or venous capillaries, which we are not able to see with CT imaging.

Vascular changes in the lungs can be studied in many different ways. Our study focuses on the blood volume discrepancies between COVID and non-COVID parenchymal areas. We included the whole vascular tree in our vascular volumes, i.e., pulmonary arteries, arterioles, capillaries, venules, and pulmonary veins, as well as bronchial vascularization to a lesser extent. Thus, any change in vascular volumes between healthy and diseased volumes could be due to changes in any compartment. Other approaches, for example, the analysis of lung perfusion changes or the segmentation of only certain types of vessels with defined diameter thresholds, are possible.

In a study including 563 chest CTA, Poletti and al. tested an automated segmentation method and found an increased total blood volume and increased volume of larger lung vessels in COVID-19 pneumonia compared to non-COVID 19 patients (35). Another study by Muriel and al. showed increased blood volumes in small vessels between 5 and 10 mm^2^ and above 10 mm^2^ (36).

In a retrospective study including 48 patients, Lang and al. found dilated distal vessels in 41 patients, with a mix of regional hyperemia in the affected zones in 13 patients and oligemia in 24 patients (37).

In a pilot study using Dual Energy CT (DECT), Si-Mohamed and al. observed increased perfusion in the affected parenchyma during the early phase of COVID-19 and decreased perfusion after 2 weeks (21).

Our study shows similar results. Our major findings are the significant difference in vascular volumes proportions between COVID (C_vasc_) and non-COVID (NC_vasc_) areas. As can be seen in Table 2 and Figure 2, C_vasc_ was systematically higher in the affected regions with a mean difference (D_vasc_) of 5% and a mean ratio (R_vasc_) of 3.7. These results can also be appreciated in the segmented volume images, such as in Figure 1 where venous congestion with increased vascular volumes are seen COVID affected areas. Such findings support the hypothesis of the presence of recruited arteriovenous shunts due to the local inflammatory state and/or local hypoxia of the lung parenchyma.

Coexistence of hypoxemia, pulmonary venous hypertension, endothelial inflammation/injury and predominantly pulmonary venous thrombosis might suggest a sequence involving selective active vasoconstriction of small-to intermediate-size pulmonary veins leading to venous microvascular congestion, blood flow stasis and thrombosis as well as pulmonary venous hypertension and hypoxemia that is exaggerated by opening arteriovenous anastomoses.

Although we put an emphasis on recruitment of pulmonary arteriovenous anastomoses, multiple other processes could co-exist and participate in the difference of vascular volumes. For example, vasoactive mediators such as mast-cells derived cytokins, angiotensin II (Ang II), vasoactive intestinal peptide (VIP), endothelin-1 (ET-1), etc. could be involved in this process (38–41). Thromboxane A2 and F2-isoprostanes are two other important vasomediators known to induce venoconstriction *via* activation of TP receptors in COVID-19 patients (42–44), It is relevant that the TP receptor blocker, ramatroban, was found in anecdotal clinical experience to relieve respiratory distress and hypoxemia in COVID-19 patients (45).

Other factors could also participate or potentialize the recruitment of vascular anastomoses, such as microthrombosis, endotheliitis or a mix of increased increased pulmonary pressure and cardiac output (32, 46, 47).

Our study is subject to several limitations. First, the number of included patients is low as we only included 10 patients; however, this was sufficient, given the statistically significant differences we found. As a consequence, we did not include additional cases. Second, the study’s retrospective nature is a potential source of bias. Third, we excluded patients presenting with dense consolidation in COVID affected regions as it resulted in over-segmentation, with an overlap that would probably result in an overestimation of the vascular volumes discrepancies between COVID and non-COVID territories. Another method should be used if one wants to assess vascular volumes in patients with more consolidation (i.e., patients in a later course of disease), which could result in interesting data for another study. Fourth, we segmented the whole vascular tree and could thus not determine whether the observed differences were more prevalent in the vessels’ capillary, venule or venous sections. Finally, segmentation thresholds were defined manually, and while we kept the same value in each patient’s left and right lung, there was interpatient variation. An artificial intelligence based automatic segmentation could provide better results, but such algorithm does not currently exist. While we focused on CT modality for this paper, further works could explore other modalities to confirm our findings, such as MRI or doppler echography.

## 5. Conclusion

Vascular volume, obtained by semi-automatic segmentation in COVID-19 pneumonia was significantly higher in areas affected by alveolar opacification than lung segments appearing normal. We found a 3.7 mean ratio of proportion of vascular volume between COVID and non-COVID affected areas. These results are consistent with other reports mentioning venous enlargement, increased lung perfusion in affected zones, and the supposed recruitment of pre-existing intrapulmonary arteriovenous shunts that could explain the discrepancies between the morphological disease severity on imaging and the clinical presentation of the patients.

## Data availability statement

The raw data supporting the conclusions of this article will be made available by the authors, without undue reservation.

## Ethics statement

The studies involving human participants were reviewed and approved by Ethics Committee of Vaud, Switzerland (project-ID 2020 -01469, 24 November 2020). Written informed consent for participation was not required for this study in accordance with the national legislation and the institutional requirements.

## Author contributions

DR and SQ: conceptualization and supervision. DR: methodology. SQ, GF, and DR: validation. A-CR and LG: investigation. GF: writing—original draft preparation. DR and CP: writing—review and editing. All authors contributed to the article and approved the submitted version.

## Funding

Open access funding by University of Lausanne.

## Conflict of interest

The authors declare that the research was conducted in the absence of any commercial or financial relationships that could be construed as a potential conflict of interest.

## Publisher’s note

All claims expressed in this article are solely those of the authors and do not necessarily represent those of their affiliated organizations, or those of the publisher, the editors and the reviewers. Any product that may be evaluated in this article, or claim that may be made by its manufacturer, is not guaranteed or endorsed by the publisher.
